# Scoping review protocol of central chronic medicines dispensing and distribution programme for widening access to medications in South Africa

**DOI:** 10.1136/bmjopen-2024-087332

**Published:** 2025-03-03

**Authors:** Olubunmi Margaret Ogbodu, Busisiwe Mrara, Olanrewaju Oladimeji

**Affiliations:** 1 Department of Anaesthesiology and Critical Care, Faculty of Health Sciences, Walter Sisulu University - Mthatha Campus, Mthatha, Eastern Cape, South Africa; 2 Department of Anaesthesiology and Critical Care, Walter Sisulu University, Mthatha, Eastern Cape, South Africa; 3 Department of Public Health, Sefako Makgatho Health Sciences University School of Science and Technology, Ga-Rankuwa, Gauteng, South Africa

**Keywords:** Chronic Disease, Drug Therapy, HIV & AIDS, Diabetes Mellitus, Type 2, Tuberculosis

## Abstract

**Abstract:**

**Introduction:**

The Central Chronic Medicines Dispensing and Distribution (CCMDD) programme, a differentiated alternative service delivery programme, initiated by the Department of Health, South Africa, allows clinically stable patients to receive chronic medication refills at the clinic-based or community-based pick-up points, offering stable patients suffering from non-communicable diseases an easy way to collect their medication. This facilitates the achievement of positive therapeutic outcomes and underscores the importance of this programme, which has resulted in decreased stigma concerns and optimising the workload for public health facilities and health workers. Therefore, this scoping review aims to explore and describe how the improved CCMDD programme has widened access to medications in South Africa in readiness for the implementation of the National Health Insurance.

**Methods and analysis:**

This scoping review will be conducted using the Arksey and O’Malley framework and further refined by the Levac framework. The review will follow a six-step approach: (1) identifying the research question, (2) identifying relevant studies, (3) studying selection eligibility, (4) charting the data, (5) collating, summarising and reporting the results and (6) consultation. A comprehensive search strategy will be developed by searching studies published between 2014 and 2024 using the following electronic databases; PubMed, Web of Science and Google Scholar. Grey literature including conference abstracts and reports will also be searched. The Preferred Reporting Items for Systematic Reviews and the Meta-Analysis for Scoping Reviews (PRISMA-ScR) will be used as a guide for this scoping review protocol. Two independent reviewers will screen identified studies’ titles, abstracts and full texts. Discrepancies will be handled by consensus or consulting a third reviewer author. Data extraction will be conducted using a standardised form. The selection of studies for the review is anticipated to be completed within 10 weeks, from 15 March to 30 May 2025, with strict adherence to the guidelines of the PRISMA-ScR checklist.

**Ethics and dissemination:**

This review, not requiring ethical approval, will inform policymakers, researchers and healthcare professionals to improve the deliverables of the CCMDD programme for all chronic conditions and ailments with a high prevalence in South Africa and identify any research gaps. We plan to disseminate our findings via a peer-reviewed journal, policy briefs, conference presentations and stakeholder engagement.

**STRENGTHS AND LIMITATIONS OF THIS STUDY**

- The review will follow a well-established scoping review methodology using the Arksey and O’Malley framework, refined by the Levac *et al* framework.
- The selection of articles will cover studies published in the last decade (2012–2024).
- The inclusion of grey literature strengthens our review by reducing publication bias and enhancing our comprehensiveness of findings.
- The study selection will include relevant studies published in English and other languages, ensuring a wide search and providing valuable insights from other countries to improve the deliverables of the Central Chronic Medicines Dispensing and Distribution programme in achieving widened access to medications.
- Studies unindexed in the consulted databases and grey literature will be omitted from the review.

## Background

 The South African National Department of Health (NDoH) launched the Central Chronic Medicines Dispensing and Distribution (CCMDD) programme, positioned within the National Health Insurance (NHI) programme in 2014 to support adherence and retention on antiretroviral therapy (ART) by enabling patients to access their medicines from contracted, community-based pick-up points (PUPs) where they live and work contributing to patient’s adherence to treatment.^1^ The CCMDD programme was implemented initially in 10 pilot NHI districts in eight provinces, excluding Western Cape Province, by the NDOH SA^2^ with the expectation that compliant and stable chronic patients in the public sector would no longer have to travel long distances or wait long hours for their medication at healthcare facilities^3^ In 2016, the NDoH began to catalyse the expansion of the programme from the initial 11 pilot districts toward a national scale to facilitate increased access to chronic medications.^1 3^ Non-communicable diseases (NCDs) kill 41 million people each year, equivalent to 74% of all deaths globally, with 77% of all NCD deaths present in low- and middle-income countries (LMICs)^4^ with the age-adjusted death rates from NCDs being nearly twice as high in LMICs as in high-income countries.^5^ Furthermore, the comorbidities of HIV and NCDs in LMICs share many common grounds that make them ideal candidates for coordinated health system approaches. South Africa, home to 7.7 million people living with HIV, and supporting the largest ART programme worldwide, with a population of 4.8 million people on ART has a growing burden of NCDs including diabetes and hypertension, accounting for 51% of mortality death rates from NCDs.^6^,^8^ Access to essential medicines remains a key element for the delivery of high-quality service and an essential part of universal coverage because it supports patient’s adherence to treatment and improves treatment outcomes.^9^ With the prevalence and rising incidence of NCDs in South Africa and the preparedness for the achievement of the 2030 Sustainable Development Goals (SDG) particularly SDG 3 which has a focus on health promotion for all, a call to integrate HIV services with NCD care, maximising resources and addressing comorbidities necessitated a keen interest in models of differentiated service delivery to ensure that health services and related medications for chronic conditions are available and accessible to those who need them, when and where they are needed most.^10 11^ The importance of health systems in delivering integrated chronic care, including increased access to medications for patients living with chronic illness, was alluded to by Vorkoper *et al.*^12^

The CCMDD programme expansion is an indication of the potential for a differentiated service delivery strategy in resource-limited settings that can be uncertain of the patients’ chronic disease condition. Globally, decentralising routine medication collection, particularly for patients living with chronic diseases who may be vulnerable to infectious diseases, has become increasingly important as health systems adjust to the threat of the COVID-19 pandemic, and the need for health systems to be resilient cannot be over-emphasised.^13^ Overall, the CCMDD no doubt has the following advantages: the convenience of picking up medications, accessibility to medications and shorter wait times associated with refill PUPs and clinical decongestion. Nonetheless, implementation challenges and contextual factors related to clinical infrastructure, medication errors, refill date inflexibility, poor communication between clinics and pick-ups regarding missed refills, lack of PUPs in rural areas and lack of patients’ data in the clinical facilities have mitigated against the full potential of the programme.^14^,^16^ The effective implementation of the CCMDD programme will ensure equal access to medications in both rural and urban areas, and this is particularly important as South Africa is in the process of implementing universal healthcare for all citizens through the National Health Insurance; therefore, an understanding of an assessment of the increased access to chronic medications due to improved CCMDD programme and identification of existing gaps such as strategies to improve the implementation of the CCMDD programme are critical.

Different authors have described similar medication delivery programmes in other resource-constrained settings in Africa which highlight improved medication adherence and increased access to chronic medications.^17^,^19^

According to the authors, in Pungoma County in Kenya, Tran *et al*^17^ described the deployment of a community-based medication delivery programme based on lessons learnt through evidence-based HIV-community-centred care strategies to design a community-based medication delivery programme for hypertensive medications. This intervention was designed based on challenges encountered by patients and negatively impacting access to chronic medications; these challenges include but are not limited to transportation costs, inability to consistently pay for medications out-of-pocket and loss of productive hours from absenteeism due to clinical visits. The community-based medication delivery programme resulted in the following benefits at the end of the study; improved self-reported medication adherence, reduced systolic blood pressure, enhanced chronic medication possession by patients and overall improved health outcomes for patients with chronic ailments.

Corroboratively, studies from Eswatini and Uganda^18 19^ revealed similar benefits of increased access to medicines and a reduction in the stockouts of essential chronic medications at health facilities. In Eswatini, optimal treatment outcomes in chronic diseases, HIV and NCDs were achieved through the deployment of an automated medication dispensing system that was patient-friendly, patient-centred, easy, convenient and quick, resulting in increased access to medications. Specifically, Uganda adopted a model similar to the CCMDD programme in South Africa as one of its new distribution approaches for lifesaving medications to improve patient outcomes and quality of life of patients using chronic medications^19^

In line with the above country-specific experiences, a systematic review by Ogungbe *et al*^20^ which examined interventions targeted at improving medication adherence among patients with cardiovascular diseases in LMICs assert that comprehensive medication adherence interventions which incorporate multiple strategies with reminders through phone calls (toll-free) and text messages, particularly customised messages to patients improved the effectiveness of the messaging service in the pick-up of medications from the PUPs.

This article outlines the protocol for a scoping review of published literature focusing on the impact of implementing the CCMDD programme in achieving widened access to chronic medications or otherwise in preparation for the National Health Insurance in South Africa; providing useful insights to healthcare practitioners, researchers, policymakers and relevant stakeholders on the benefits and lessons from the implementation of the CCMDD programme as an innovative model of integrated service delivery to expand access to chronic medications in South Africa.

## Methods and analysis

### Protocol design

**Figure 1:**
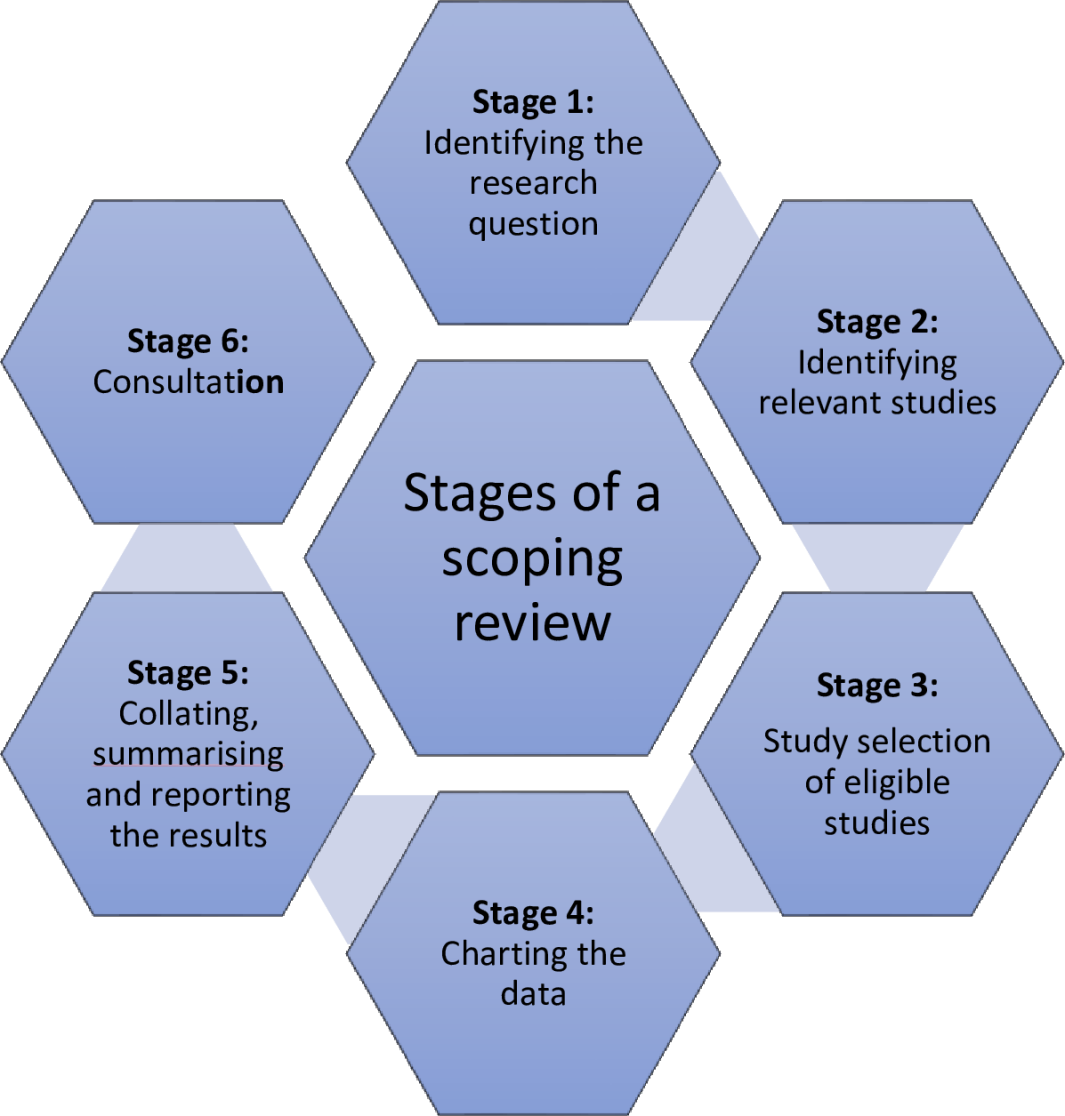
Six stages of conducting a scoping review (Arksey H, O’Malley L. Scoping studies: towards a methodological framework. *Int J Soc Res Methodol* 2005;8:19–32. 43).

The methodology of this scoping review is derived from the approach developed by Arksey and O’Malley^21^ and further refined by Levac *et al*.^22^ The review will be conducted in six stages as shown in figure 1. Our approach will also incorporate methodology developed by the Joanna Briggs Institute.^23^ The protocol for this review is not registered with PROSPERO due to its current non-acceptance of scoping reviews. We adhered to the Preferred Reporting Items for Systematic Reviews and Meta-Analysis extension for Scoping Reviews (PRISMA-ScR) in crafting the protocol^24^ and guiding the presentation of our results.^25^ In addition, we compared the protocol to previously published protocols while adhering to the same guidelines and incorporating BMJ’s additional expectations.

### Stage 1: identifying the research question

We developed the research question using the technique suggested by Arksey and O’Malley and further refined by Levac *et al*. The main research objective is ‘To describe how the CCMDD program has widened access to chronic medications in South Africa, in preparation for the National Health Insurance’. Our multidisciplinary team comprises a pharmacist and two physicians; all three are public health practitioners.

The research subquestions are

1. To identify the enablers of the CCMDD programme in South Africa.
2. To identify the challenges associated with implementing the CCMDD programme in widening access to chronic medications.
3. To describe the strategies for achieving an improved CCMDD programme, of benefit to South Africans living with chronic diseases.

This study will use the Population, Intervention/Exposure, Comparison, Outcomes and Timeline (PICOT) format (table 1) to align the study selection with the research question. In this study’s applied PICOT framework, the population under consideration includes patients on chronic medications who collect medications from accredited health facilities or private PUPs. The intervention or exposure of interest is CCMDD. There is no comparison group in this framework. The outcomes being assessed are the enablers and challenges of effective CCMDD implementation contributing to widened access to chronic medications for patients on the CCMDD programme. The timeline for this study will span 10 weeks from 15 March 2025 to 30 May 2025, and eligible studies will be from 2012 to 2024. The implementation phase of the NHI pilot in South Africa began in 2012, hence the selected timeline of 2012 to assess the benefits and lessons from the programme implementation.

**Table 1:** PICOT framework for assessing the Central Chronic Medication Dispensing and Distribution (CCMDD) programme for widening access to medications for National Health Insurance implementation in South Africa

Criteria
P - Population	Patients on chronic medications who collect medications from accredited health facilities or private pick-up points
I - Intervention	CCMDD or differentiated drug delivery and distribution programme
C - Comparison	No comparison group
O - Outcome	Enablers of effective CCMDD and differentiated drug delivery and distribution programme implementationChallenges of effective CCMDD implementation
T - Timeline	2012–2024

### Stage 2: identifying relevant studies

We will comprehensively search electronic databases (PubMed, Web of Science and Google Scholar) and grey literature sources for studies published between 2014 and 2024. The following three databases will be used: PubMed, Web of Science and Google Scholar. An initial exploratory search strategy based on the PICOT framework will be developed on PubMed to determine some relevant terms to chronic medication delivery programmes and chronic ailments. A second search strategy will be developed using the most relevant Medical Subject Headings terms while some keywords will be searched both in the title, abstract and subject headings on Web of Science and Google Scholar. The precise, full strategies for all databases used are included (in a table) as an online supplemental file. Lastly, reference lists from the retrieved reviews on related topics will be used as an additional source for searching for additional articles in a snowball approach. To locate potentially relevant grey literature, targeted searches will be conducted on dissertations/theses and conference abstracts (using Pharmaceutical Conference Abstracts and Conference Proceedings). The search terms will include a combination of keywords related to CCMDD, NHI, PUPs, NCDs, HIV and ART. We will also hand-search the reference lists of included studies and review articles for additional studies, including websites such as the WHO and United Nations.

### Stage 3: study selection of eligible studies

To ensure a rigorous selection process, we will employ the PIOT framework (table 1) as a guide for the title and abstract screening. Additional eligibility criteria will be implemented to guarantee the selection of studies that are pertinent to our research question. It is anticipated that the selection of studies for the review will be completed within 10 weeks, and this process will adhere to the guidelines provided by the PRISMA-ScR checklist.^25^ Once all identified records have been extracted from all databases, duplicates will be eliminated using the EndNote V.X9 software. The entire selection process will be executed using a reviewing platform, specifically Rayyan^26^ to facilitate collaboration between reviewers, maintain transparency and streamline the reviewing process. Any discrepancies between the two reviewers during the screening and selection stages will be resolved through discussion and, if needed, consultation with a third reviewer to reach an agreement.

### Inclusion criteria

Titles and abstracts will be screened for eligibility by two independent reviewers based on the following inclusion criteria:

1. Study conducted on drug delivery programmes implemented in low-resource settings in Africa to improve patient adherence and clinical outcomes for patients with chronic diseases.
2. The study focuses on patients on chronic medications who collect medications from accredited health facilities or private PUPs.
3. The study reports on how medication delivery programmes like the CCMDD have been implemented in South Africa and other African countries, highlighting the enablers and challenges of chronic medication delivery programme implementation.
4. Published studies between 2012 and 2024. The same two reviewers will assess full-text articles for eligibility. Any discrepancies will be resolved through discussion and consultation with a third reviewer. Should any ambiguity arise during the first stage of screening, the full text will be obtained for additional clarification.

### Exclusion criteria

Studies will be excluded if they have any of the following characteristics:

1. Studies from non-African countries.
2. Studies focusing on non-vulnerable participants.
3. Studies where full-text articles could not be obtained.

### Types of studies

We will consider experimental (randomised or non-randomised), observational studies (longitudinal, cross-sectional) and mixed-methods

### Quality assessment

Quality assessment is described as a necessary component of scoping studies as it enables reliable research evidence to be disseminated to a wider audience in a way that is useful to practice and policymaking and for future researchers;^27^ therefore, this component is included in the selection of studies to be charted. The PRISMA-ScR checklist, a tool that supports comprehensive reporting of methods and findings, based on the PRISMA statement and checklist, JBI methodological guidance and helps to improve the quality of scoping reviews by enhancing transparency and uniformity in reporting will guide this study. The Newcastle-Ottawa Scale^28^ will be used to assess the quality of cross-sectional studies found during the review. By employing a systematic and comprehensive approach, this scoping review aims to establish a thorough synthesis of the evidence of community-based medication delivery programmes such as the CCMDD for improved medication adherence and increased access to chronic medications in South Africa, highlighting the gaps and areas for future research. No language restrictions will be applied to the search. Additionally, the bibliography details of inclusion research and relevant reviewed studies will be manually sourced to pick out any more research that may have been overlooked in the electronic sourcing.

### Stage 4: charting the data

Building on our research objectives outlined in the PICOT framework (table 1), we will proceed to chart and analyse the data from the selected studies. Data will be extracted using a predefined charting form (table 2), which is designed to capture critical details about each study. It is important to note that while our PICOT framework guided the scope and focus of our study, this charting form now enables us to systematically organise and analyse the data, pinpointing key areas such as the study design, setting, population and key findings.

**Table 2:** Data charting form

1	Lead author
2	Year of publication
3	Title of study
4	Aim of study
5	Study design
6	Study setting/country
7	Study population
8	Age group
9	Sample size (number of participants)
10	Eligibility criteria
11	Intervention
12	Study outcome
13	Has improved CCMDD widened access to chronic medications in preparation for National Health Insurance in South Africa?
14	Enablers of improved delivery of the CCMDD programme in widening access to chronic medications in all the provinces in South Africa
15	Challenges associated with the delivery of the CCMDD programme in widening access to chronic medications in all the provinces in South Africa facilitators
16	Recommendations from the study

CCMDDCentral Chronic Medicines Dispensing and Distribution

### Stage 5: collating, summarising and reporting the results

**Figure 2:**
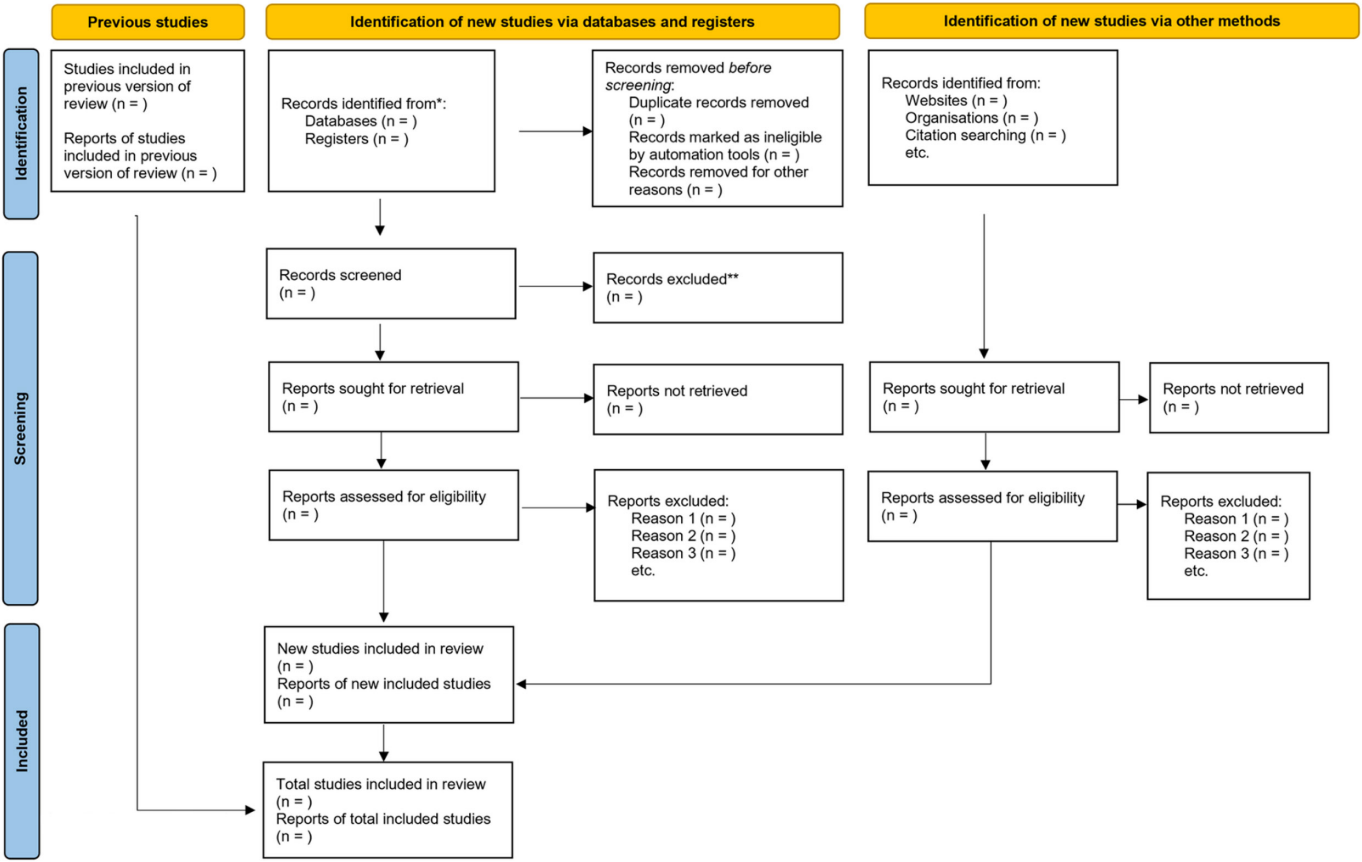
Preferred Reporting Items for Systematic Review and Meta-Analysis extension for Scoping Reviews (PRISMA ScR) flowchart for eligible study selection (Tricco AC, Lillie E, Zarin W, O’Brien KK, Colquhoun H, Levac D, *et al* (2018). PRISMA Extension for Scoping Reviews (PRISMA-ScR): Checklist and Explanation. *Ann Intern Med*, 169 (7), 467–473).

The data will be analysed using appropriate methods for each type of study included in the scoping review. For qualitative studies, we will employ thematic content analysis to identify themes related to how CCMDD has widened access to chronic medications in South Africa. This will be any notable quotes or narratives from participants that shed light on their experiences, perceptions or attitudes towards the CCMDD programme. This process will involve the following steps: Two trained members of our research team will independently code the data. To enhance the reliability and validity of our analysis, the data will be independently dual-coded. Any discrepancies in the coding process will be resolved through discussion and consensus, involving a third researcher if necessary. Themes will be developed using an inductive approach, where themes will emerge organically from the data. This will allow for a rich, grounded understanding of the research topic. While our approach is primarily inductive, the overarching PICOT framework (table 1) will provide a broad conceptual guide for our theme development. To facilitate efficient and rigorous analysis, we will use ATLAS.ti, a software tool designed for managing, coding and analysing qualitative data. This tool will allow us to organise our data systematically and visualise relationships between themes, enhancing the depth and thoroughness of our analysis. For quantitative studies, we will extract information as stated in the data charting form. Our goal is to answer the research question and achieve the study’s overarching objective. We will present our findings in a comprehensive report, including a narrative summary alongside tables, graphs and diagrams to visually represent key results as shown in figure 2.

### Stage 6: consultation

We will consult with stakeholders, including researchers, policymakers and representatives from populations of interest particularly the healthcare workers operating the CCMDD programme like the pharmacists, pharmacy assistants and professional nurses, and users of the CCMDD programme to validate and interpret the findings of this scoping review. While stakeholders have not directly contributed to the design of this review, we are in the process of establishing channels for their involvement in interpreting our findings. For instance, we plan to organise stakeholder meetings or workshops where we will present our findings and invite feedback and discussion. We also plan to conduct one-on-one consultations, either in-person or virtually, depending on the stakeholder’s location and preference. To access these stakeholders, we will leverage our existing networks and partnerships within the public health sphere. We are committed to ensuring that the consultation process is inclusive and respectful, recognising the expertise and relevance that each stakeholder brings. We believe that their input will be invaluable in helping us identify research gaps, interpret our findings within the proper context and make relevant recommendations and suggestions for future research.

## Discussion

HIV and non-communicable disease care both require ongoing attendance at appointments at health facilities, adherence to tests and medications and healthy living and self-care. Therefore decentralising medication pick-up services and moving care to the community rather than requiring individuals to travel long distances to health facilities can play critical roles in supporting treatment adherence in HIV care and non-communicable disease care.^7^ The treatment adherence assertion has been supported by studies that reveal and describe the CCMDD programme, as an innovative, differentiated service delivery strategy that demonstrates the potential of widening access to medications in resource-limited settings.^3 9 11 13^ Despite the commendations for the benefits of the CCMDD, a critical review of challenges including late delivery of medications, patients receiving collection messages late, incorrect medications issued to the patients and lack of PUPs in rural areas must be conducted to overcome such challenges to have a more robust implementation experience at all CCMDD health facilities and PUPs.

The implementation of health system strengthening measures such as the Central Chronic Medication Dispensing and Distribution in resource-constrained settings underscores the importance of improving health outcomes by widening access to medications for patients.^11^ Studies from other resource-constrained African settings reveal that having a central, community-based medication delivery programme holds great promise for achieving increased access to chronic medications with improved patient outcomes, medication adherence and enhancing the quality of life of patients.^18^,^20^ In conclusion, this scoping review will provide a summary of how the CCMDD programme has improved and widened access to chronic medications in South Africa, highlighting the enablers and challenges of the programme. Additionally, the scoping review will identify gaps in the existing literature and suggest future research topics. The findings of the scoping review will be used to develop targeted strategies to overcome the identified challenges of the programme in widening access to chronic medications given its implication for national health insurance implementation in South Africa. The study’s findings will be peer-reviewed and published in a scientific journal, and the abstract will be presented at local and international conferences. The review’s findings will be distributed to health facilities participating in the CCMDD programme, health ministries and policymakers to improve the deliverables of the CCMDD programme for chronic ailments and inform policy directions on how improved CCMDD will expand access to chronic medications in South Africa. Overall, the effective implementation of the CCMDD programme should ensure equal access for all patients to their medications, in both rural and urban areas in South Africa, contributing to the achievement of universal health coverage for all.

### Patient and public involvement

This is a scoping review protocol. No participants were interviewed. Patients and/or the public were not involved in the design, conduct or reporting of this research; however, relevant stakeholders will be consulted as part of the dissemination plans of this research.

### Ethics and dissemination

Ethical approval is not required as this is a scoping review of the existing literature. We acknowledge that ensuring our research reaches those it affects most is a critical step in conducting ethically sound and impactful research. In addition to publication in a peer-reviewed scientific journal and presentations at academic conferences and webinars, we intend to take multiple approaches to ensure our research findings reach the target communities and stakeholders. Our dissemination strategies will be customised based on the needs and characteristics of the target communities. For instance, we plan to organise community meetings or workshops in collaboration with health planners at the local health departments and community-based organisations, where we can present our findings in an understandable and accessible manner.

## supplementary material

10.1136/bmjopen-2024-087332 online supplemental file 1
