# Supplementary material for: Scoping review protocol of central chronic medicines dispensing and distribution programme for widening access to medications in South Africa
Source: BMJ Open. 2025 Mar 3;15(3):e087332. doi: 10.1136/bmjopen-2024-087332 (PMC11877235; doi:10.1136/bmjopen-2024-087332)
Supplement: online supplemental file 1 [file bmjopen-15-3-s001.docx]

Precise database search strategy for CCMDD manuscript in BMJ Open journal

| **Database** | **Search Strategy** | **Keywords** |
| --- | --- | --- |
| **PubMed** | “Chronic medication distribution and delivery program” (MeSH Terms) OR “Community-based medication delivery program in Africa” (All Fields) OR “Widened access to chronic medications in Africa” (All Fields) OR: differentiated distribution and delivery of chronic medicines at health facilities in South Africa” (Heading) OR “delivery of chronic medicines to patients in South Africa” OR “improved medication adherence due to delivery of chronic medications in Africa” OR “absenteeism from work and loss of employment (All Fields) OR “distribution and delivery of ARTs to people living with HIV/AIDS” (Heading) OR “ challenges of access to chronic medications for NCDs in Africa” OR “pilot implementation of the NHI in South Africa; lessons and benefits” OR “ lessons from Africa on community-based distribution and delivery of chronic medicines to patients” AND (2012:2024) | CCMDD, NHI, PUPs, NCDs, HIV and ART |
| **Web of Science** | TITLE-ABS-KEY (Chronic medication distribution and delivery program in South Africa*”) OR TITLE-ABS-KEY (“Lessons from Africa on community-based distribution and delivery of chronic medicines to patients*”)) OR (Community-based medication delivery program in Africa*” OR “Widened access to chronic medications at public health facilities in Africa*” OR “experiences of patients at pick-up points when receiving chronic medicines at public health facilities in Africa*”)) OR “The implementation of the CCMDD program to improve access to chronic medicines in African settings, lessons learned?” OR “ is there evidence for the continued utilization of the community-based dispensing and delivery of ART and NCD medications to patients in Africa?” AND (“2012 – 2024”). | CCMDD, NHI, PUPs, NCDs, HIV and ART |
| **Google Scholar** | ((Facilitators of community-based medication delivery program for chronic medicines in South Africa” OR “interventions that improve patient adherence to the use of chronic medicines” OR “enablers of an effective chronic medicine distribution and delivery program in resource-constrained African countries”)) OR “challenges faced by service users of the CCMDD program in South Africa” AND (2012 – 2014). | CCMDD, NHI, PUPs, NCDs, HIV and ART |
