# Supplementary material for: Scoping review protocol of central chronic medicines dispensing and distribution programme for widening access to medications in South Africa
Source: BMJ Open. 2025 Mar 3;15(3):e087332. doi: 10.1136/bmjopen-2024-087332 (PMC11877235; doi:10.1136/bmjopen-2024-087332)
Supplement: Supplementary file 2 [file bmjopen-2024-087332.reviewer_comments.pdf]

PEER REVIEW HISTORY

BMJ Open publishes all reviews undertaken for accepted manuscripts. Reviewers are asked to complete a checklist review form and are provided with free text boxes to elaborate on their assessment. These free text comments are reproduced below.

ARTICLE DETAILS

Title (Provisional)

SCOPING REVIEW PROTOCOL OF CENTRAL CHRONIC MEDICINES DISPENSING AND DISTRIBUTION PROGRAMME FOR WIDENING ACCESS TO MEDICATIONS IN SOUTH AFRICA

Authors

OGBODU, OLUBUNMI Margaret; Mrara, Busisiwe; Oladimeji, Olanrewaju

---

VERSION 1 - REVIEW

**Reviewer**	**1**
**Name**	**Shortland, Diane**
**Affiliation**	**University of Birmingham Edgbaston Campus, school of medicine and dentistry**
**Date**	**15-Jul-2024**
**COI**	**No competing interests**

I think this scoping review is a worthwhile piece of research and will provide a valuable contribution to the field. It will be useful to know of similar initiatives in other countries and settings where this goal of medication delivery may have been implemented and its positive and negative outcomes, benefits and issues. Pooling collectively all experiences of such programmes will be useful for this project but also in many other contexts, rural communities and outreach initiatives. The authors give a good overview of dissemination of findings and the intention to enter consultation with stakeholders to invite feedback and discussion is valuable. Direct patient involvement would further shed light on the actual first-hand experience of using the service and could lend to a follow-up study whereby service users are interviewed for their unique perspectives of the programme.

Whilst in the main this protocol is well prepared and covers all aspects one would expect to find in a scoping review procedure, I would encourage the authors to be sure of their choice of scoping review, as opposed to systematic review, and reflect this clearly in their wording. A scoping review is ideal for mapping out the scope and coverage of research done on a given topic when the emerging evidence is still unclear and more specific questions cannot be posed (Peters et al 2020 JBI Manual of Evidence Synthesis). In their inclusion criteria the

authors refer to inclusion point no.3 where they give inclusion to a study if it ‘reports on how the CCMDD has improved and widened access’. What about studies on medication delivery programmes that may have failed to achieve improved access? Perhaps a clearer more open-ended wording would suit and support the scoping review rationale here.

In terms of methodology, the use of Arksey & O’Malley framework is relevant and is pleasing to see it has been incorporated by more recent additions of Levac et al and the Joanna Briggs Institute. Revisions of scoping procedures however, have also noted the value of quality assessment (Daudt, Mossel & Scott 2013 BMC Medical Research Methodology p.6).

Scoping reviews by nature tend to be broader in their collection of studies and traditionally it is a systematic review that would appraise a narrower selection of studies but give valuable quality assessment. It is disappointing the authors do not intend to include any form of quality assessment of studies. Including a critical appraisal in this scoping review would allow for the achievement of both objectives: a broad scoping to reduce risk of missing studies but also an appraisal of studies giving information on quality of methodology within studies. Since the effectiveness of implementation of medication delivery programmes is of key importance, it is paramount to know that such potential study results are reliable and valid, and based on strong execution. In their discussion, the authors comment that a ‘critical review of challenges… must be conducted’. Without any form of critical assessment, findings can surely only be summarised? If time does not allow for full quality assessment, perhaps an additional section on the data charting form could include a reference to overall methodological standard? I believe this would add to the overall objectives and produce really worthwhile findings.

To delve into more detail on the methodology of the proposed review, there are a couple of areas where I believe greater clarity is needed. The proposed databases to be used differ in the abstract to the methods section. It is therefore unclear what the full list of databases will be. I also think it would be useful to include a search strategy of keywords, perhaps as an example table, since it is unclear exactly what the keywords will be or how the authors arrived at these choices, i.e. via an initial search of limited databases perhaps? For the purposes of reproducibility and transparency, these areas would benefit from a more precise explanation.

With some minor revisions, I feel this protocol for a scoping review will be a beneficial exercise that will give further light to health facilities offering greater medicine collection access.

**Reviewer**	**2**
**Name**	**Atuhairwe, Irene**
**Affiliation**	**Stellenbosch University, Public Health**

Date **09-Dec-2024**

COI **None**

The protocol is well written I suggest the following to improve it; currently it is not easy to clearly make out the objectives of the study I suggest that the authors include a section on the objectives clearly stating the objectives.

I also suggest to get a variety of studies and evidence, the authors should consider studies outside South Africa.

With the current technology, language should no longer be a barrier and so the authors should consider removing the language restrictions to English only.

Under the steps for conducting a scoping review, the authors included consultation after the review is done to hear their feedback on the results, I would suggest this consultation is conducted incoperated earlier from formation of the research questions through out the process instead of doing it only at the end.

VERSION 1 - AUTHOR RESPONSE

Comments from Reviewer 1

Reviewer 1: I think this scoping review is a worthwhile piece of research and will provide a valuable contribution to the field. It will be useful to know of similar initiatives in other countries and settings where this goal of medication delivery may have been implemented and its positive and negative outcomes, benefits and issues. Pooling collectively all experiences of such programmes will be useful for this project but also in many other contexts, rural communities and outreach initiatives.

Response: We sincerely value your perspective on the inclusion of similar initiatives of medication delivery in other countries and settings, benefits, outcomes and issues. We agree with you on this in enriching the narratives, lessons, and experiences from different countries. Therefore, we have included in the background section, studies and lessons from other African countries namely Kenya, Eswatini, and Uganda, and a systematic review of medication delivery for chronic illnesses in low and medium-income countries.

Reviewer 1: The authors give a good overview of dissemination of findings and the intention to enter consultation with stakeholders to invite feedback and discussion is valuable. Direct patient involvement would further shed light on the actual first-hand experience of using the service and could lend to a follow-up study whereby service users are interviewed for their unique perspectives of the programme.

Response: Thank you for your candid feedback, we appreciate it. As this is a scoping review of existing literature, direct patient involvement is not intended. However, we acknowledge that we must ensure that our research reaches those it affects most is crucial in conducting sound and impactful research therefore our initial submission that we will enter into consultation with stakeholders which will now include service users of the CCMDD service in South Africa to invite feedback and unique perspectives of the implementation of the CCMDD programme.

Reviewer 1: Whilst in the main this protocol is well prepared and covers all aspects one would expect to find in a scoping review procedure, I would encourage the authors to be sure of their choice of scoping review, as opposed to systematic review, and reflect this clearly in their wording. A scoping review is ideal for mapping out the scope and coverage of research done on a given topic when the emerging evidence is still unclear and more specific questions cannot be posed (Peters et al 2020 JBI Manual of Evidence Synthesis).

In their inclusion criteria, the authors refer to inclusion point no.3 where they give inclusion to a study if it 'reports on how the CCMDD has improved and widened access'. What about studies on medication delivery programmes that may have failed to achieve improved access? Perhaps a clearer more open-ended wording would suit and support the scoping review rationale here.

Response: We are grateful for your feedback on the protocol and the scoping review procedure. Our scoping review protocol will be guided by the Preferred Reporting Items for Systematic Reviews and the Meta-Analysis for Scoping Reviews (PRISMA-ScR) checklist to help prepare and report the scoping review protocol and not the Systematic Reviews and Meta-Analyses Protocols (PRISMA-P) guidelines as reported in the original manuscript. We apologise for not communicating this effectively in our initial submission; our study is a scoping review protocol, not a systematic review. Our article outlines the protocol for a scoping review of published literature focusing on the impact of implementing the CCMDD programme in achieving widened access to chronic medications or otherwise in preparation for the National Health Insurance in South Africa; providing useful insights to healthcare practitioners, researchers, policymakers and relevant stakeholders on the benefits of improved CCMDD as an innovative model of integrated service delivery to expand access to chronic medications in South Africa.

In line with the above, we have reviewed the inclusion criteria under methodology to include all studies that focus on medication delivery programmes for chronic ailments, this will give room to learn lessons from widened access or otherwise from such programmes.

Reviewer 1: In terms of methodology, the use of Arksey & O'Malley framework is relevant and is pleasing to see it has been incorporated by more recent additions of Levac et al and the Joanna Briggs Institute. Revisions of scoping procedures however, have also noted the value of quality assessment (Daudt, Mossel & Scott 2013 BMC Medical Research Methodology p.6). Scoping reviews by nature tend to be broader in their collection of studies and traditionally it is a systematic review that would appraise a narrower selection of studies but give valuable quality assessment. It is disappointing the authors do not intend to include any form of quality assessment of studies. Including a critical appraisal in this scoping review would allow for the achievement of both objectives: a broad scoping to reduce risk of missing studies but also an appraisal of studies giving information on quality of methodology within studies. Since the effectiveness of implementation of medication delivery programmes is of key importance, it is paramount to know that such potential study results are reliable and valid, and based on strong execution. In their discussion, the authors comment that a 'critical review of challenges… must be conducted'. Without any form of critical assessment, findings can surely only be summarised? If time does not allow for full quality assessment, perhaps an additional section on the data charting form could include a reference to overall methodological standard? I believe this would add to the overall objectives and produce really worthwhile findings.

Response: We appreciate the robust feedback on the need to include a quality assessment of studies. In alignment with the assertion by Daudt, Mossel and Scott 2013 on the importance of conducting a quality assessment of studies as it enables reliable research evidence to be

disseminated to a wider audience in a way that is useful to practice and policymaking and for future researchers, we have incorporated a quality assessment of studies, under stage 3: selection of eligible studies, in the manuscript.

Reviewer 1: To delve into more detail on the methodology of the proposed review, there are a couple of areas where I believe greater clarity is needed. The proposed databases to be used differ in the abstract to the methods section. It is therefore unclear what the full list of databases will be. I also think it would be useful to include a search strategy of keywords, perhaps as an example table, since it is unclear exactly what the keywords will be or how the authors arrived at these choices, i.e. via an initial search of limited databases perhaps? For the purposes of reproducibility and transparency, these areas would benefit from a more precise explanation.

Response: Thank you for your insightful feedback. We apologise for the mix-up, the proposed databases in in the abstract and methods sections are now properly aligned. Three databases will be used; PubMed, Web of Science and Google Scholar. An initial exploratory search strategy based on the PICOT framework will be developed on PubMed to determine some relevant terms to chronic medication delivery programmes and chronic ailments. A second search strategy will be developed using the most relevant Medical Subject Headings (MeSH) terms while some keywords will be searched both in the title, abstract and subject headings on Web of Science and Google Scholar. The precise, full strategy strategies for all databases used are included (in a table) as a supplementary file with this revised submission. Lastly, reference lists from the retrieved reviews on related topics will be used as an additional source for searching for additional articles in a snowball approach.

For reproducibility and transparency as requested, we have provided a more precise explanation for the search strategy to be used for the study.

Reviewer: 2

Ms. Irene Atuhairwe, Stellenbosch University, Seed Global Health

Comments to the Author:

Reviewer 2: The protocol is well written I suggest the following to improve it; currently it is not easy to clearly make out the objectives of the study I suggest that the authors include a section on the objectives clearly stating the objectives.

Response: Thank you for commending the protocol write-up, this is greatly appreciated and encouraging. As suggested, we have included a section in the manuscript, stating clearly the objectives.

Reviewer 2: I also suggest that the authors consider studies outside South Africa to obtain a variety of studies and evidence.

Response: Thank you for this great perspective of yours. We have included studies from other resource-constrained countries such as Kenya, Uganda, and Eswatini in the revised manuscript.

Reviewer 2: With the current technology, language should no longer be a barrier and so the authors should consider removing the language restrictions to English only.

Response: Yes, we align with your submission to remove the language barrier with current technology. Therefore, we have rephrased the inclusion criteria so that all languages dealing with the subject matter will be considered in the review.

Reviewer 2: Under the steps for conducting a scoping review, the authors included consultation after the review is done to hear their feedback on the results, I would suggest this consultation is conducted and incorporated earlier from the formation of the research questions throughout the process instead of doing it only at the end.

Response: A scoping review is designed to provide an overview of the extent and nature of a body of literature and our article outlines the protocol for a scoping review of published literature focusing on the impact of implementing the CCMDD programme in achieving widened access to chronic medications or otherwise in preparation for the National Health Insurance in South Africa. The study will provide useful insights to healthcare practitioners, researchers, policymakers, and relevant stakeholders on harnessing the benefits and lessons from the implementation of CCMDD as an innovative model of integrated service delivery to expand access to chronic medications in South Africa. As this is a scoping review protocol, we will be working with published literature to map evidence on the effectiveness of chronic medication delivery in resource-constrained settings. However, based on your recommendation and the comments from Reviewer 1, we have stated in our manuscript that we will enter into consultation with stakeholders which will now include service users of the CCMDD service in South Africa to invite feedback and unique perspectives of the CCMDD programme.

Dear Editor, with the above explanations, we have provided point-by-point responses to the suggested reviews and edits from the Editor and the reviewers. Kindly let us know if you find them in order and we are available to provide any additional information if required.

VERSION 2 - REVIEW

**Reviewer**	**1**
**Name**	**Shortland, Diane**
**Affiliation**	**University of Birmingham Edgbaston Campus, school of medicine and dentistry**
**Date**	**24-Jan-2025**
**COI**	

I have enjoyed reading this revised manuscript and am pleased to see all comments have been taken on board and amended accordingly to ensure this protocol reaches a high standard of proposed execution.

The strengths & limitations section would benefit from more effective shorter bullet points, but the search strategies are now clearly visible with keywords highlighted to enable reproducibility, and the objectives are clearly stated as three questions to be answered. I am pleased to see that service users will now be part of the dissemination process as well as stakeholders, and that the introduction now includes useful service delivery experiences of other countries, which gives a more insightful background to this review and why it’s being

done, having set the scene more comprehensively. I am also delighted to see a change in criteria to now include all studies that focus on medicine delivery programmes, not just those with positive outcomes. This will give a greater picture and truer presentation of what is known on the topic of previous study trials. Again it is encouraging to see that quality assessment is now intended to take place too, resulting no doubt in much more useful findings that give detail on thorough methodology that will provide crucial information in offering this service moving forward.

I am now much more confident that this study will be a useful exercise in taking this area of knowledge further and will also be conducted to a high research standard.
